# Challenges in implementing model-based phase I designs in a grant-funded clinical trials unit

**DOI:** 10.1186/s13063-017-2389-2

**Published:** 2017-12-28

**Authors:** Eleni Frangou, Jane Holmes, Sharon Love, Naomi McGregor, Maria Hawkins

**Affiliations:** 1 Centre for Statistics in Medicine, Nuffield Department of Orthopaedics, Rheumatology and Musculoskeletal Sciences, University of Oxford, Windmill Road, Oxford, OX3 7LD UK; 2 Oncology Clinical Trials Office (OCTO), Department of Oncology, University of Oxford, Old Road Campus Research Building, Roosevelt Drive, Oxford, OX3 7DQ UK; 3 CRUK MRC Oxford Institute for Radiation Oncology, Gray Laboratories, University of Oxford, Old Road Campus Research Building, Roosevelt Drive, Oxford, OX3 7DQ UK

**Keywords:** CRM, Continual reassessment method, Design, Phase I, Model-based, Dose-finding

## Abstract

**Background:**

For a clinical trials unit to run its first model-based, phase I trial, the statistician, chief investigator, and trial manager must all acquire a new set of skills. These trials also require a different approach to funding and data collection.

**Challenges and discussion:**

From the statisticians’ viewpoint, we highlight what is needed to move from running rule-based, early-phase trials to running a model-based phase I study as we experienced it in our trials unit located in the United Kingdom. Our example is CHARIOT, a dose-finding trial using the time-to-event continual reassessment method. It consists of three stages and aims to discover the maximum tolerated dose of the combination of radiotherapy, chemotherapy, and the ataxia telangiectasia mutated Rad3-related inhibitor M6620 (previously known as VX-970) in patients with oesophageal cancer. We present the challenges we faced in designing this trial and how we overcame them as a way of demystifying the conduct of a model-based trial in a grant-funded clinical trials unit.

**Conclusions:**

Although we appreciate that undertaking model-based trials requires additional time and effort, they are feasible to implement and, once suitable tools such as guiding publications and document templates become available, the design and set-up process will be easier and more efficient.

## Background

Phase I clinical trials comprise a significant part of a clinical trials unit’s portfolio, particularly in the cancer community, as they test the first administration of a single agent or combination of agents to humans. These trials aim to identify the maximum tolerated dose (MTD), which is the dose that does not cause dose-limiting toxicities (DLT) above the target toxicity level (TTL). They are either algorithm-based, such as the 3 + 3 or model-based, such as the continual reassessment method (CRM).

Over the past several decades, most phase I studies have used a 3 + 3 design or one of its modifications. The 3 + 3 enrols cohorts of three patients at a time. The first cohort is treated at a starting dose that is considered to be safe. If at least one of these patients experiences a DLT, a second cohort of three patients is assigned to the same dose level. Otherwise, the second cohort is treated at the next, higher dose level. The dose escalation proceeds until at least two of six patients (33%) experience a DLT at a given dose level. The dose level just below this level is then recommended for further exploration. The many modifications of the 3 + 3 design have relaxed some of its rules, but all forms make dose-escalation decisions by considering the previous three to six patients.

In contrast, model-based designs estimate the dose-toxicity relationship with a statistical model, and use this model to allocate patients to doses and find the MTD. Data from all enrolled (completely and partially followed up) patients are used to continuously update the dose-toxicity model. This model guides every dose-allocation decision and therefore results in a more accurate estimate of the MTD. An advantage of model-based designs is that more patients are treated near the MTD without exposing them to additional risk [1].

One of the modifications to the initial CRM design, published in 2000, is the time-to-event continual reassessment method (TiTE-CRM) [2]. This design allows late-onset toxicities to be accounted for without fully observing a patient before recruiting the next, and uses all accumulated information to recommend each dose. As we do not have to wait until complete follow-up, the overall trial duration is shortened. This is a step change for radiotherapy trials, where toxicity follow-up is often 3–6 months. Having to pause recruitment while the current cohort is fully followed up makes traditional methods infeasible and from a trial management perspective also cumbersome.

CHARIOT is the first trial in our grant-funded clinical trials unit using a TiTE-CRM design. Until this time, we had been unable to convince clinicians to use a model-based design but the benefits in this trial due to the late-onset toxicities outweighed any resistance. In this article, we describe the development of the UK trial CHARIOT as experienced by the trial statisticians; from initial discussions with the chief investigator (CI) to securing funding and writing the trial protocol and statistical analysis plan. We discuss the design and characteristics of CHARIOT (see Trial example), present the challenges we faced and difficulties we overcame to build an efficient and robust model framework (see Challenges/features), and summarise our learning (see Discussion and Conclusions).

## Trial example

CHARIOT is a single-arm open-label, phase I dose-escalation trial and is the first trial examining the combination of radiotherapy, chemotherapy, and the ataxia telangiectasia mutated Rad3-related (ATR) inhibitor M6620 (previously known as VX-970) in patients with oesophageal cancer. The aim is to identify the maximum tolerated schedule associated with no more than a predefined TTL. CHARIOT consists of three stages, each with a different TTL. Although each stage is characterised by its own set of inclusion/exclusion criteria, number of treatment schedules, and treatment duration, we used the same approach to choose the trial specifications, including the DLTs and TTLs, in all three.

**Fig. 1:**
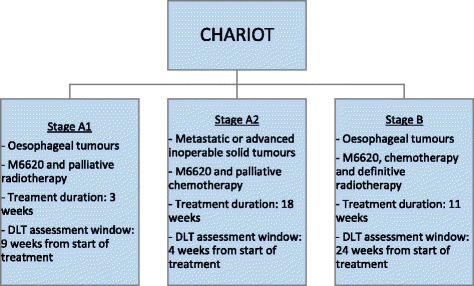
Set-up of CHARIOT – this schema outlines the main characteristics of the three stages in CHARIOT

Figure 1 outlines the main characteristics of each stage. Stage A1 will address patients with oesophageal cancer receiving palliative radiotherapy. There will be three frequencies of administering M6620 and two doses, giving six treatment schedules of interest. The dose and frequency of radiotherapy will remain constant. The setting for stage A2 will be patients with any solid tumour receiving only chemotherapy. Two doses of M6620 will be tested at weekly and twice-weekly frequencies, giving four treatment schedules.

Once we have enough information from stages A1 and A2, the M6620 will be tested in stage B. It will be administered in combination with cisplatin, capecitabine, and radical radiotherapy to identify the MTD, which will be taken forward in future phase II studies. The patients in stage B will have oesophageal cancer amenable to radical treatment. Six treatment schedules will be tested. The frequency of administration of M6620 differs across the different schedules. The dose of M6620 at each administration will not change and will be informed by the results of stages A1 and A2.

We assume that as the schedule number increases, toxicity and efficacy increase as well (monotonicity assumption). Modelling only the schedule and toxicity relationship via a dose-toxicity curve is therefore sufficient. As the DLT assessment windows in each stage are long (Fig. 1), a TiTE-CRM design will be used in all three stages to find the optimal treatment schedule.

As a model-based design, TiTE-CRM allows us to tailor the trial to the CI’s requirements. For example, as CHARIOT is the first trial exploring these lines of treatment, the CI requests to treat the first three patients on the lowest treatment schedule and observe them for the entire DLT window before recruiting the next patient. This will provide insights into the profile of the radiotherapy and M6620 combination. As we also want dose escalation to be fairly rapid, we will use cohorts of one and make dose-allocation decisions every time a patient is recruited.

The trial design ensures:

- schedule allocation for each newly recruited patient after the first three patients are fully followed-up;
- no treatment schedule skipping on escalation for untried schedules;
- no restriction on treatment schedule de-escalation;
- the treatment schedule assigned will be that estimated to be closest to, but not above, the MTD;
- a safety stopping rule will restart the trial using a lower dose of M6620 or terminate when sufficient evidence suggests that the lowest schedule is overly toxic. We will consider the lowest schedule to be too toxic if, given all available data, there is a high probability (95%) that the DLT rate is greater than the TTL;
- early stopping for success when an adequate number of patients has been treated at a dose level where we define “success” to mean stabilisation of the MTD estimate;
- recruitment slots will be used to ensure enough information is accumulated to inform the best assignment of the treatment schedule to a patient. The clinical trials unit will inform recruiting sites when a slot becomes available and the window in which treatment should begin. For example in A2, as soon as the patient starts treatment the next slot can be made available, maximising recruitment whilst allowing an appropriate review period for the previous patient. Further, to enable recruitment, two slots will be available at any point when there is more than 4 weeks since the previous patient started treatment;
- stage A1 and stage A2 to run in parallel.

Each escalation decision will be made by the trial management group (TMG), consisting of the CI, the principal investigators, the trial manager, and the trial statisticians, and will be based on the recommendation of the TiTE-CRM model and the accumulated experience with the treatment. If the TMG disagrees with the model’s recommendation to escalate or a stopping rule has been met, an independent data safety and monitoring committee will review the decision. This independent committee will include at least one model-based design expert.

## Challenges/features

The challenges faced during the set-up of CHARIOT are not presented in a strictly hierarchical order; going back and forth was part of the learning process.

### Communication within the trial team

Communication between the statisticians, the CI and the rest of the clinical trial unit’s staff was key during the early stages of the set-up process. Meetings were required to ensure the defined treatment schedules were nested, the monotonicity assumption was satisfied, and stopping rules for success and safety were established. The CI needed to specify the skeleton, which is the level of toxicity she might expect to see on each treatment schedule. This is a challenging task, which she was facing for the first time. She initially found the task daunting, but reviewing the literature, discussing the issue with fellow clinicians, and trying to put toxicity expectations into numbers resulted in reasonable prior probabilities. Deciding on an acceptable TTL was another new task facing the trial management team. Other discussions included how to use data from the first two stages to inform the final stage, when to start the final stage, and how to shorten the entire trial duration.

### Statistical programming

CRM and TiTE-CRM have been implemented in various statistical software packages. However, code was not available to accommodate all of the trial’s characteristics, such as a pause in recruitment after the first three patients; a delay between recruitment and assigning a dose level in stage B, as all patients will receive the same treatment for the first 6 weeks; different time-to-event toxicity distributions; dose-escalation rules that do not allow overdosing based on the accumulated information; and early stopping rules. Two trial statisticians therefore independently developed programs for executing and simulating the trial and for processing their results using R and OpenBugs. Writing our own code helped us to understand the underlying mechanisms of the method. Writing two versions allowed us to validate the statistical programs before executing on real data, which is a requirement of our unit’s standard operating procedures. Due to the complexity of the code, additional time was allowed for debugging.

We decided important trial properties and characteristics, like the skeleton, escalation rules, stopping rules, and sample size, by combining clinical expertise with results from a range of simulation scenarios.

### Simulations

Simulations played an integral role in designing CHARIOT. They facilitated understanding of the mechanics and behaviour of the model under a range of circumstances and led the selection of specific design characteristics appropriate for CHARIOT. We simulated trials with different early stopping rules, maximum sample sizes, DLT distributions, and dose-escalation rules. The simulation properties are given in more detail in Table 1.

**Table 1:** Design characteristics for simulations

Design characteristic	Specifics
Stopping rule	6, 8, 10 patients on a dose, no early stopping
Maximum sample size	Stages A1 and A2: 15, 20, 25, Stage B: 20, 25, 30
Distribution of toxicities	Uniform, triangular with the mode half-way through the dose-limiting toxicity window
Dose escalation	With or without overdosing allowed
Dose allocation	No dose skipping when escalating but no restrictions in de-escalating
Accrual rate (patient/weeks)	1/4, 1/6, 1/8

For each set of design rules, we checked the properties of the trial under different toxicity distributions, which were chosen to reflect realistic and extreme scenarios.

**Fig. 2:**
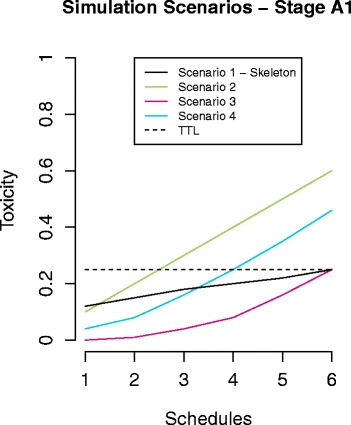
Simulation scenarios explored for stage A1 – the *black solid line* shows the skeleton (also explored as a scenario). The *coloured solid lines* define the dose toxicity curve that we simulated data from. The *black dashed line* shows the target toxicity level (TTL)

Figure 2 presents the scenarios explored for stage A1. We ran 1000 simulations for each toxicity distribution and design rule. The OpenBugs model ran for 10,000 iterations for each simulation, of which 1000 were set as burn-in. Simulations of each scenario gave us operating characteristics such as the number of times each schedule was selected as the MTD, the average number of patients treated on each schedule, the sample size, and the trial duration. We then used these operating characteristics to guide our choice of a design with the best properties.

Discussing simulation results with non-statistical team members helped them to understand the CRM design. By simulating single trials and working through them step by step, we were able to show how dose decisions would be made during the trial.

### Trial management

The TiTE-CRM requires maximum data capture to make the most accurate recommendation. This requires participating sites to provide real-time data for every participant just prior to the TMG meeting. To facilitate this, we developed a dedicated case report form (CRF) requiring only data essential to the execution of TiTE-CRM code. If tight timelines dictate a TMG to be held without much notice, data can be communicated through the telephone and the code can be executed during the meeting. As the information being presented to the TMG is novel and differs considerably from traditional “3 + 3” escalation procedures, the trial management team decided to hold a mock TMG meeting using dummy data prior to the recruitment of the first patient.

To enable the clinicians to plan for the TMG, as treatment generally starts on a Monday, we plan to hold a TMG meeting every Wednesday which will occur only if a participant has been recruited. This would allow enough time to prepare the prescription and to deal with any issues arising from the dose recommendation being rejected by the TMG.

Radiotherapy DLTs can have a late onset and we want to assign dose with maximum accumulated data on each patient. It is, therefore, critical to report all DLTs promptly. To achieve this we decided to report all DLTs using the serious adverse event (SAE) reporting system which is familiar and robust. This will ensure that the CI and TMG will know about the occurrence of a DLT within 24 hours of the site being aware.

As total drug requirements could not be known in advance, we produced two forecasts. The first assumes that all treatment schedules are recommended and the maximum number of patients is required to complete the study. The second forecast is based on the simulation results. Both forecasts were provided to the pharmaceutical company supplying M6620 to show drug requirements and cost under two plausible scenarios.

### Funding

Funding for statistics work was required for two periods of the trial design process. Considerable work was needed before the grant application, and this statistical time was funded from local resources. Funding for statistics input during the trial set-up was requested in the grant application. Although the trial design was received well by the funding body, they questioned “why funding for a statistician is needed for a phase I trial”. We were fortunate to be able to fund the statistical input from local resources.

In future grant applications we plan to move as much of the statistical input for design into the trial set-up period, and therefore within a grant, as possible. A trial design must be named when applying for a grant, but time can be allocated in the application to finalise the design parameters. We also note that there are trial design funding opportunities via the UK Joint Global Health Trials scheme (UK Department for International Development (DFID), Medical Research council (MRC), National Institute for Health Research (NIHR), Wellcome Trust) and the US National Institutes of Health (NIH). As we design more such studies we build up experience and template documents to speed the set-up process.

### Manpower and lead time

As CHARIOT was our unit’s first trial using TiTE-CRM, between two and three statisticians were assigned to work on the trial, familiarise themselves with the method through self-study, courses, and seminars, and, ultimately, ensure that the TiTE-CRM design could meet the CI’s expectations. External advice was also sought through the Methodology Advisory Service for Trials (MAST) of the MRC Hubs for Trials Methodology Research [3].

## Discussion

When we set out to design our first TiTE-CRM trial, our unit only had experience in running A + B trials. We therefore faced a steep learning curve. The planned trial requires three stages, has a long toxicity period, and tests treatment schedules rather than a single drug at different doses. We discovered that discussing the TTL and prior toxicity probabilities of each schedule during design meant that the whole trial team started the trial more aware of the expected toxicity than would usually be the case in a phase I trial.

Support from experienced statisticians via the MRC Methodology Hubs and MAST [3] was essential in setting up this trial. As interest in implementing model-based trials is growing, the NIHR is trying to facilitate the use of CRM by showing how the barriers to these designs can be overcome [4].

The European regulatory guidance on first-in-man trials [5] does not dictate a design [6–9] and our ethics committee and the funder were both supportive of the planned design.

As a grant-funded clinical trials unit, we were fortunate to be able to fund the design stage of our first TiTE-CRM trial from local resources. As we did not know how much statistical detail should be included in a model-based trial grant application, preparing the grant application took longer than anticipated. We ran lots of simulations and had many meetings with the trial management team about the design, so that we had a full trial design when we applied for the grant. However, we have learnt that many of these details could have been finalised during trial set-up, after funding had been received. In future grant applications, we will leave the finer details of the design for later in the process and will instead simply describe the trial in broad and lay terms.

With the benefit of a deeper understanding of TiTE-CRM, we now realise that investing time in testing some aspects of the design, such as pausing recruitment after the first cohort to look at safety, does not affect the model’s results or performance. However, code is still needed to test different escalation rules. We appreciate that first trials of any design are particularly hard to fund and believe that future model-based trials will not be as time-consuming for our statistics team. Table 2 summarises issues that cost us valuable time and suggestions on how to deal with them. This information is not available in the literature and is learning that we are keen to share with the community.

**Table 2:** Issues we faced while designing CHARIOT and suggestions on how to deal with them, based on what we have learnt and wished we knew before starting the trial design

Area	Issue	What to do
Software	All trial details must be programmed	Use existing software to get as close as possible
Funding	A great deal of statistical input is required before the grant is awarded	− This issue is more pronounced for the first few model-based trials that a clinical trials unit undertakes. Once the process is well-known, code will be in place and templates will have been designed for text in the protocol and statistical analysis plan − Consider stating the design without much detail in the grant application. Leave some aspects of the design to be refined within the grant, during trial set-up
Data management	Trial requires fast (e.g., within 24 hours) and accurate data entry	− Have required data collated on a single case report form − Accept data during teleconferences (as is done in 3 + 3 designs) and run the model during the teleconference − Classify all dose-limiting toxicities as serious adverse events to ensure prompt reporting
Statistical knowledge	− Trial statisticians need to check they have made good decisions − Enhance trial statistician learning	– MAST (UK-based) – Consult colleagues with experience in such designs through a registered clinical trials unit network

*MAST* Methodology Advisory Service for Trials

We held a well-received course for non-statisticians on how TiTE-CRM works to engage them with the design and to facilitate future trial meetings. After 10 minutes of theory, we ran a trial with the attendees as patients using the statistical software we had developed. Toxicity events were represented by taking a ball out of a bag and checking the colour. As well as giving attendees a good idea of how the trial would work, it also showed that the model could run in real time and quickly generate dose-escalation guidance. The attendees also took part in dosing decisions for patients in the simulated trial, which is valuable practice for the real decisions that the TMG will need to make during the actual trial.

Only 6% of early phase trials currently use model-based methods [10]. To improve uptake, clinical trials units, particularly those without prior experience, need finance for statistician salaries, patience and greater input from their clinicians and more practical guidance in the literature. At the moment, lack of published guidance means clinical trial units are having to each reinvent the wheel when designing their first model-based trial. We need more publications that explain trials in detail [11–16], more papers that lead a trial team through the practical steps of designing a CRM trial, and template and exemplar protocols and statistical analysis plans for teams to start from.

## Conclusions

Model-based designs are complex but we found they are feasible to set up in an academic trials unit. Suitable guidance on how to implement these studies is needed to make the process of trial design and set-up easier and more efficient. We hope that this article describing our experience and learning in designing a model-based trial will be a first step in building the needed literature base to support trial teams.

## Abbreviations

ATR: Ataxia telangiectasia mutated Rad3-related
CI: Chief investigator
CRF: Case report form
CRM: Continual Reassessment Method
DFID: Department for International Development
DLT: Dose-limiting toxicities
MAST: Methodology Advisory Service for Trials
MRC: Medical Research Council
MTD: Maximum tolerated dose
NIH: National Institutes of Health
NIHR: National Institute for Health Research
SAE: Serious adverse event
TiTE-CRM: Time-to-event continual reassessment method
TMG: Trial Management Group
TTL: Target toxicity level
